# Within-Colony Variation in the Immunocompetency of Managed and Feral Honey Bees (*Apis mellifera* L.) in Different Urban Landscapes

**DOI:** 10.3390/insects6040912

**Published:** 2015-10-29

**Authors:** R. Holden Appler, Steven D. Frank, David R. Tarpy

**Affiliations:** 1 Department of Entomology, North Carolina State University, Raleigh, NC 27695, USA; E-Mails: rholdenappler@gmail.com (R.H.A.); sdfrank@ncsu.edu (S.D.F.); 2 W.M. Keck Center for Behavioral Biology, North Carolina State University, Raleigh, NC 27695, USA

**Keywords:** urbanization, pollinator populations, honey bees, encapsulation response, phenoloxidase

## Abstract

Urbanization has the potential to dramatically affect insect populations worldwide, although its effects on pollinator populations are just beginning to be understood. We compared the immunocompetency of honey bees sampled from feral (wild-living) and managed (beekeeper-owned) honey bee colonies. We sampled foragers from feral and managed colonies in rural, suburban, and urban landscapes in and around Raleigh, NC, USA. We then analyzed adult workers using two standard bioassays for insect immune function (encapsulation response and phenoloxidase activity). We found that there was far more variation within colonies for encapsulation response or phenoloxidase activity than among rural to urban landscapes, and we did not observe any significant difference in immune response between feral and managed bees. These findings suggest that social pollinators, like honey bees, may be sufficiently robust or variable in their immune responses to obscure any subtle effects of urbanization. Additional studies of immune physiology and disease ecology of social and solitary bees in urban, suburban, and natural ecosystems will provide insights into the relative effects of changing urban environments on several important factors that influence pollinator productivity and health.

## 1. Introduction

Behavioral flexibility and division of labor are often accredited to the extreme ecological success of social insects [1]. This “social physiology” often involves high variability of worker phenotypes within colonies that enable them to be buffered from perturbations in the local environment [2]. Western honey bees, *Apis mellifera*, are a model study system within the social insects because they are highly amenable to experimental manipulation and exhibit extraordinary phenotypic plasticity. A colony consists of a single mated female (the queen) with tens of thousands of workers (her daughters) that perform all non-reproductive tasks, and their intricate division of labor is influenced, at least in part, to their extreme genetic diversity as a consequence of hyperpolyandry (multiple mating) of the queen [3].

While group living has been highly successful ecologically, it also introduces certain costs that can challenge insect societies. Perhaps most notably is the increased likelihood of harboring and enabling the spread of disease-causing agents [4], placing a premium on different mechanisms of physiological immunity (at the individual level) and “social immunity” (at the colony level; [5,6,7]). A suite of individual-level immune responses, including encapsulation and phenoloxidase activity, are important for insect health and have been well elucidated in honey bees (e.g., [8,9]). Encapsulation is a defense against large pathogens and parasitoids whereby hemocytes bind to the invader’s surface triggering the melanin pathway, blocking access to the insect’s internal environment while also producing cytotoxic intermediates [10,11]. The aforementioned melanin pathway is mediated by phenoloxidase, the precursor of which (prophenoloxidase) is produced within specialized hemocytes [12,13]. Though individual immune systems contribute to the overall health of the colony [6,14], they are metabolically expensive to maintain and function appropriately without causing self-harm [15,16]. Nonetheless, the high intra-colony genetic diversity in honey bees is mirrored by the high variation in physiological immunity at the colony level [17].

Honey bees are not only an important research model, they are also the most common pollinators in most agroecosystems worldwide [18,19], and non-commercial gardens and wildflowers benefit from both managed and feral (non-managed) honey bees for pollination. Honey bees are typically managed for pollination services and honey production, and in doing so beekeepers actively control for parasites and pathogens, manipulate colony size, and routinely move their beehives. In contrast, feral honey bees—those found nesting in cavities of trees, buildings, or other confined spaces [20,21,22]—are not subject to honey harvesting, disease treatments, artificial hive population enhancement, swarm control, hive size augmentation, or other management practices. Without human intervention, feral honey bees are potentially less-buffered against stressors like starvation, parasites and pathogens, water scarcity, and limited colony space. With the recent decline of the managed honey bee population in the U.S. (reviewed in [23,24]) and major losses in feral honey bees since the introduction of the mite *Varroa destructor* [25], it is vital to understand what environmental influences can affect the health of feral honey bees and the ecosystem services they provide (c.f. [9,26,27]).

Urbanization—the process of converting natural or agricultural land into suburbs and cities—is an increasingly important agent of environmental change worldwide. As cities continue to expand in both geographic size and population, they will have greater environmental influence through processes like urban sprawl [28,29,30] and pollution [31,32,33]. Such changes can have dramatic, yet disparate effects on the organisms that inhabit the urbanized regions [29]. However, we have little understanding of how insect immunity is affected by urbanization, particularly in important pollinators such as honey bees. Urban areas have more impervious surface that reduces the area and connectivity of floral resources. This may force bees to forage farther from their hive, increasing foraging costs, and could reduce energy invested in immune functions [34]. On the other hand, floral diversity can be greater in urban than natural areas [35,36,37,38] and more diverse pollen sources have been shown to bolster the metabolically expensive immune system [39]. Impervious surfaces also increase the temperature contributing to the “heat island effect” [40]. High temperatures could decrease bees and colony health [41,42] by directing resources away from individual processes (like immune function) towards the collection of water for evaporative cooling. Thus while urban environments have many attributes linked to impaired health and weakened immunocompetence, other features may be beneficial to honey bees, negating or even surpassing the deleterious effects.

Our overall objective was to determine the degree to which urbanization and active honey bee management affect the immunocompetence of individual bees. We predict that increasing urbanization negatively affects honey bee immunity. Moreover, we predict that managed honey bees will have muted immune responses compared to feral bees because we assume that feral honey bees are more exposed to their local environments and therefore the effects of urbanization.

## 2. Materials and Methods

### 2.1. Colony Location and Landscape Analysis

We tested 11 feral colonies and 12 managed (beekeeper) colonies between May and October of 2013 across an urban gradient in a 50-mile radius around Raleigh, NC (USA). All colonies were from unique locations (*i.e.*, not from the same apiary). Managed colonies were maintained by hobbyist beekeepers contacted through local beekeeping organizations. We selected colonies had been established in a single geographic location for at least one month, and all colonies had been established for at least one year and thus had survived the winter. Feral colonies were those naturally established in trees or structures without any human management. We located feral colonies through hobbyist beekeepers and the citizen-scientist website *Save the Hives*^©^ (2013; www.SaveTheHives.com), a user-submitted tool for tracking feral honey bee colonies. These colonies were located in nest cavities inside the branches or trunks of trees or within the walls of buildings, and the duration of their occupancy was unknown but was at least 6 months from its initial report. Thus, like all managed colonies, all had also survived the previous winter.

**Figure 1:**
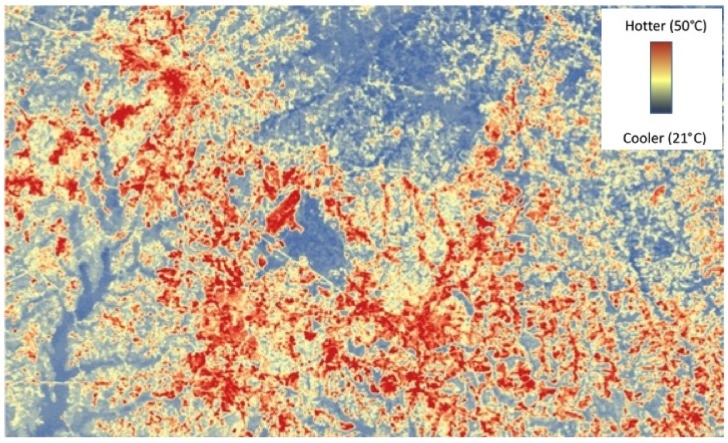
Relative temperature (UM-RH) over Raleigh, NC, and the surrounding area. Values determined from readings on 18 August 2007 (see [45] for methods).

We assigned each colony an urban location (rural, suburban, or urban) based on a visual assessment of the local habitat, based on housing density and nearby landscape. To verify these categories with more quantifiable measurements of urbanization, we analyzed each location using ArcMap 10.1 (Ersi Inc., Redlands, CA, USA) to generate values of impervious surface and average relative heat (Figure 1) up to 3 km radius surrounding the colony, corresponding to the accepted maximum foraging radius of honey bees [43]. We used two variables to measure the level of urbanization shown to affect the physiology and survival of other insects: percent impervious surface and relative surface temperature (see [44,45]). Percent impervious surface was measured as the sum total percent impervious surface constructed from the percentage of the middle range for each impervious surface group [46]. Surface temperatures were obtained from data generated from Meineke *et al.*, 2013 [45]. The average “relative heat” is a general comparison between cooler and warmer sites, rather than specific temperatures that the colonies experienced.

### 2.2. Sampling

We visited each colony and collected 50–80 foraging workers with a sweep net as they entered or exited their hive. We transported bees in a cooler to the laboratory at North Carolina State University in Raleigh, NC. Transport time for all bees was less than one hour. Since we could not access the inner combs of the feral colonies to sample younger “hive” bees, we did not take internal samples from the managed colonies to remain consistent in our comparisons. In the laboratory, we placed the bees into a −20 °C freezer until they were sufficiently immobile to handle. We placed 10–20 live bees in Plexiglas cages (10 cm × 10 cm × 7 cm) in Percival incubators set to 34 °C with a 0:24 h L:D photoperiod (see [47]). We kept the incubators dark to minimize variation due to time of day when assays would be conducted. We also provided the bees with 33% sucrose solution *ad libitum.* The bees acclimated to the incubator for at least one hour but no more than 24 h prior to assays.

### 2.3. Encapsulation Response

We tested all collected specimens for encapsulation analyses. We removed the bees from the incubator and cold anesthetized them in a −20 °C freezer and kept them on ice until processed. Encapsulation can be measured by inserting foreign objects into the hemocoel of an insect [11,48]. We inserted a roughened nylon thread probe (0.176 mm diameter) into the bee abdomen using a sterile technique [8]. To promote a complete immune response [49], we dipped the probes into lipopolysaccharide (LPS) just prior to insertion [15,48]. We returned the probed bees to the incubator cages for 3.5 h after which bees were cold anesthetized and the probes were removed with forceps at precisely 4 h post-insertion [8]. We slide-mounted the encapsulated probes along with a non-inserted “blank” and stored the slides in a −20 °C freezer until further analysis.

**Figure 2:**
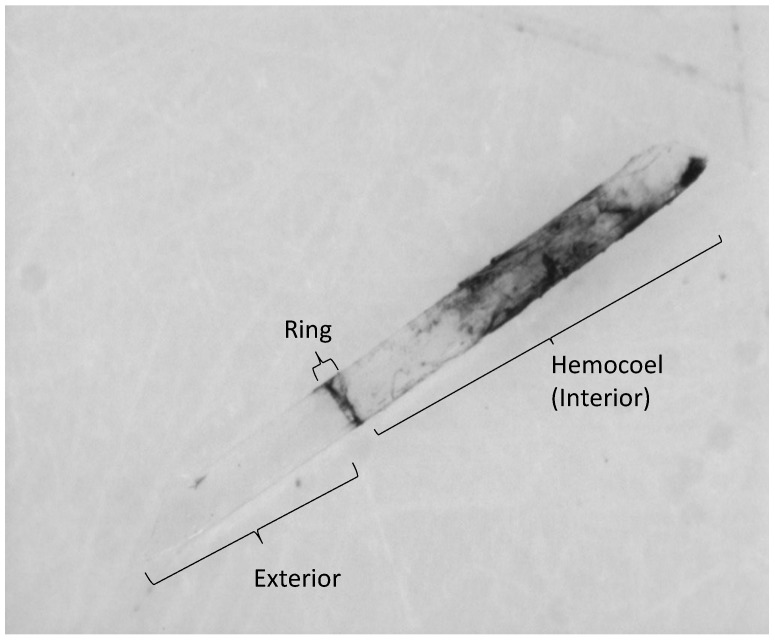
The encapsulation around a pseudo-parasite probe 4 h post-insertion. Three primary regions are identified: “exterior”, defined as the portion that remained outside of the hemocoel; “ring”, defined as the junction point between the external and internal environments of the bee; and “interior”, defined as the portion fully within the hemocoel. Encapsulation data was analyzed for the entire inserted portion (ring and interior) and the ring alone.

To quantify the encapsulation response we created monochrome 256-bit images of the probes with the software program ISCapture (v2.0, Scienon Technology, Princeton, NJ, USA). We produced images from three different orientations (to help control for shadows and light) on obverse and reverse sides of each inserted probe and control blank. We then measured each composite image for the mean grey value of the probe using ImageJ (U.S. National Institutes of Health, 1.46r; [8]). We recorded grey values for three regions on each probe: the “ring” formed at the junction at the point of insertion, the hemocoel portion alone, and the entire portion that was inserted into the hemocoel (Figure 2). We produced a composite score from the mean of the six images for each probe and subsection (e.g., three obverse ring images and three reverse ring images) to be used for individual-level analyses. We then averaged these scores to create a single colony score that would be used for future analyses.

### 2.4. Phenoloxidase Activity

We prepared hemolymph extracts from the thorax of each specimen that was analyzed for encapsulation response after the probe was removed. We prepared hemolymph extracts by grinding the thoraxes of individual bees in 300 μL of cold honey bee saline. We centrifuged the rough extracts at 1300 g for 10 min at 4 °C (Eppendorf Centrifuge 5804 R, 15 amp version), and transferred the liquid portion to a new tube. We stored the extracts at −80 °C to stop uncontrolled phenoloxidase activity and disrupt prohemocytes [8]. After at least 24 h, we thawed the extracts on ice and then vortexed them for 3 s. We centrifuged the tubes at 16,260× *g* for 15 min at 4 °C, then moved the supernatant to microcentrifuge tubes for short-term (less than 1 week at −20 °C) or long-term storage (more than 1 week at −80 °C).

We used the standard L-DOPA method to quantify phenoloxidase (PO) activity. Prior to thawing hemolymph extracts on wet ice, we prepared L-DOPA solutions from 6 mg/mL crystalline L-DOPA (Cayman Chemical Company, Ann Arbor, MI, USA, cat. No. 13248) in dH_2_O [50]. We vortexed the solution for 15 min and then stored the solution in a refrigerator to reduce degradation. We transferred 20 μL of hemolymph extracts (honey bee saline for blanks) into wells of a 96-well spectrophotometer-capable plate that contained 135 μL dH_2_O and 20 μL PBS (pH 7.4; Fisher Scientific Gibco^®^, Pittsburgh, PA, USA, cat. No. 10010-023), performed in triplicate. We added 5 μL of α-chymotrypsin solution (1 mM Tris-HCl, 2 mM CaCl_2_, 0.5 mg/mL crystallized α-chymotrypsin in dH_2_O; MP Biomedicals LLC, Santa Ana, CA, USA, cat. No. 152272) to each well, and incubated the plate at room temperature for 5 min. We then added 20 μL of L-DOPA solution to each well. As phenoloxidase is the rate-limiting step in theconversion of L-DOPA to dopachrome, phenoloxidase activity can be determined by measuring the change in absorbance by dopachrome over a 50 min period. We measured raw phenoloxidase activity as V_max_, determined from the slope during the linear phase of the reaction (KCJunior v1.22, Bio-Tek, Winooski, VT, USA). We also performed a standard Pierce^®^ BCA protein assay (Thermo Scientific, Dallas, TX, USA, cat. No. 23225) to produce a second measurement of phenoloxidase activity that would control for the hydration state of each individual, V_max_/(protein). Phenoloxidase analyses included seven feral colonies and 10 managed colonies.

Phenoloxidase activity was calculated in two ways. The established approach is to measure the V_max_ of the enzymatic reaction that forms dopachrome and to divide this value by the protein concentration in an attempt to account for hydration state (V_max_/(protein)). Additionally, we considered V_max_ alone because of the high (and very consistent) levels of soluble protein from entire thorax extractions.

### 2.5. Statistical Analyses

We conducted separate analyses for different radii for each variable of urbanization (impervious surface and relative heat) at 100, 200, 300, 500, 1000, 1500, 2000, 2500, and 3000 m. The strongest signals for impervious surface was 3 km and for relative heat was 1.5 km, and thus all analyses for these measures use the values at these distances from each colony. We then used one-way ANOVA to compare the measures of urbanization among the three urban categories.

We analyzed the individual-level measurements of encapsulation and phenoloxidase activity for feral and managed bees with respect to the three zones of urbanization (rural, suburban, and urban) using a two-way ANOVA using colony as a random variable (JMP Pro 11, SAS Institute, Cary, NC, USA). Ring encapsulation and V_max_ values were already normally distributed and therefore not transformed, while the grayness of the entire inserted portions of the probes and standardized PO activity values were natural-log transformed to normalize. All statistics were conducted with α = 0.05 and reported errors of SEM unless otherwise noted.

## 3. Results

### 3.1. Study Sites

We found highly significant differences among the three study zones for impervious surface (*F_2_*_,*36*_ = 52.0, *p* < 0.0001) and relative heat (*F_2_*_,*36*_ = 32.5, *p* < 0.0001), and in the predicted direction. Urban sites had significantly higher percentages of impervious surface (26.1% ± 1.41%) compared to suburban (18.3% ± 1.62%) and rural sites (5.2% ± 1.56%). Correspondingly, rural sites were significantly cooler (25.9 ± 0.19 °C) than suburban (27.2 ± 0.22 °C) and urban sites (28.1 ± 0.19 °C), as these two measures of urbanization are highly correlated (*r^2^* = 0.84, df = 35, *p* < 0.0001). Thus we were able to accurately categorize the urbanization zones of the studied colonies.

### 3.2. Encapsulation Response

**Figure 3:**
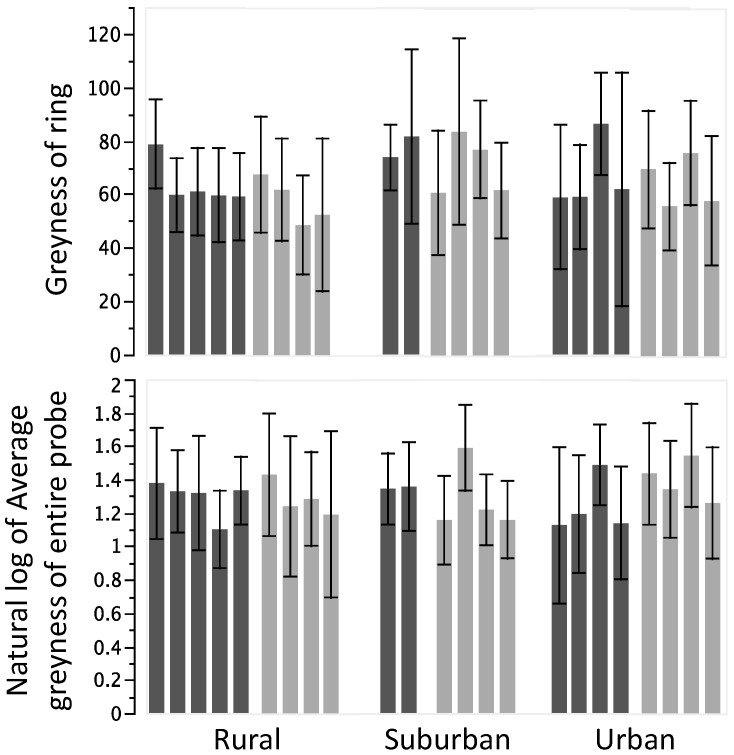
Bar graphs of each colony (mean ± SD) for measures of encapsulation response. Feral colonies (dark gray) and managed colonies (light gray). (**Top**) Encapsulation response at the “ring” portion of the probe alone; (**Bottom**) Encapsulation response of the entire probe.

Analyses of encapsulation response did not show any significant differences associated with colony type (feral *vs.* managed) or urban location (Figure 3). For the grayness of the ring portion of the probe, there were no differences between managed and feral bees (*F_1_*_,*234*_ = 1.25, *p* = 0.28) and marginal differences among colonies in rural, suburban, or urban areas (*F_2_*_,*234*_ = 3.42, *p* = 0.05) with suburban colonies being significantly higher than rural colonies and urban colonies being intermediate. Similarly, there were no differences between colony type (*F_1_*_,*234*_ = 0.36, *p* = 0.55) and locations (*F_2_*_,*234*_ = 0.04, *p* = 0.96) for the entire probe. Overall, management and urban location accounted for only 9.5% of the total variation, thus 90.5% of the phenotypic variation in encapsulation was seen among different individuals within colonies.

### 3.3. Phenoloxidase Activity

**Figure 4:**
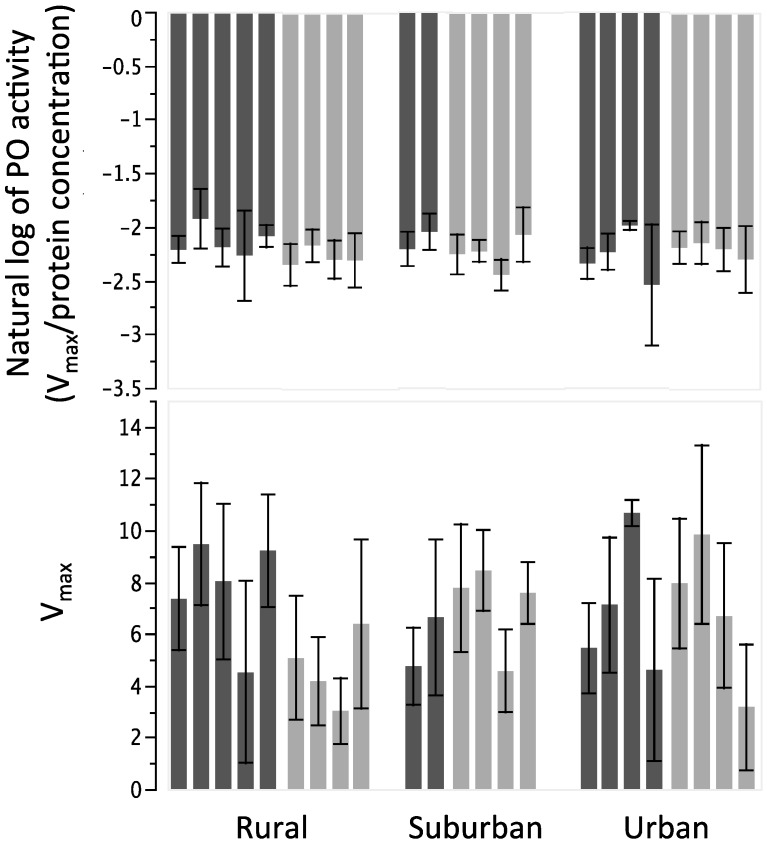
Bar graphs of each colony (mean ± SD) for measures of phenoloxidase activity. Bars plotted as in Figure 3. (**Top**) Phenoloxidase activity as measured by V_max_/(protein); (**Bottom**) Phenoloxidase activity as measured by V_max_ alone.

Neither colony type nor urbanization were significantly different for phenoloxidase activity (Figure 4). Standardized phenoloxidase activity, measured by V_max_/(protein), was not effected by colony type (*F_1_*_,*234*_ = 0.91, *p* = 0.35; Figure 4), and there was no effect of urbanization (*F_2_*_,*234*_ = 0.20, *p* = 0.82). Results when phenoloxidase activity is measured by V_max_ alone were similar to those from V_max_/(protein) (Figure 4), with no significant relationships of management type (*F_1_*_,*234*_ = 0.88, *p* = 0.36) or urban site (*F_2_*_,*234*_ = 0.16, *p* = 0.85).

## 4. Discussion

In this study, we measured the immunocompetence of honey bees in areas with different levels of urbanization. We characterized urbanization through measurements of the average relative heat and impervious surface that bees may experience within their foraging range. Individually, both urbanization measurements may represent specific biological challenges that the bees must face (e.g., heat can induce water stress, increased metabolic rates; impervious surface can increase habitat fragmentation, vegetation loss) in addition to any other general factors of urbanization (e.g., aerial particulate matter, pollutants). We were unable to associate a strong significant signal of urban location with the immune responses of honey bees. As such, our results do not support the prediction that bees in more urbanized areas have weaker immune responses. In addition, we reject the hypotheses that feral bees have stronger immunocompetence than managed bees, or that feral bees are more affected by urbanization than managed bees. While it could be argued that our measures of encapsulation are insufficient proxies for honey bee immunity, we believe that they are viable analogues of encapsulation [51,52] and that coagulation and wound healing are important measures of immunocompetence [53]. These standard measures, however, may not be robust enough to capture potential subtle or negating effects of urbanization, thus additional fine-scale measures of immunity may be warranted. We should also note that variation in worker age, longevity of the colonies, and genotype likely add to the variation in these immune phenotypes.

It remains unclear whether V_max_/(protein) or V_max_ alone best represent phenoloxidase activity, as neither measurement showed any association between relative temperature or impervious surface and phenoloxidase activity. Poikilotherms obtain much of their heat from the external environment, so that their metabolic processes are generally quickened in higher temperatures. However, there are myriad processes and tradeoffs that can occur within individual bees. Other studies have found that increasing temperature can cause higher [54,55] or lower [56] phenoloxidase activity in insects. Additionally, Suwanchaichinda and Paskewitz [57] showed that *Anopheles gambiae* adults had lowered encapsulation rates with increasing temperatures. Response to increasing or decreasing temperature depends on the actual temperature reached, the magnitude and rate of the change, and background temperature conditions.

Our urban study sites had an average impervious surface cover of 26.1%, which is low compared to other cities such as New York, NY, USA (61%), Los Angeles, CA, USA (52%), Chicago, IL, USA (59%), and Boston, MA, USA (48%), with an average of 41.3% [58]. Thus, the range and magnitude of impervious surface cover and the consequent heat, fragmentation, and other stresses may be less and require more precision or sampling to detect. Moreover, other factors such as nutrition and pollution can affect phenoloxidase activity in different ways. Karl *et al.* [56] found decreased phenoloxidase activity in nutritionally deprived *Bicyclus anynana* larvae, while those that were well provisioned had increased phenoloxidase activity with increasing temperature [55]. Water within urban settings can contain environmental contaminants and pollutants [59] that could affect the immune system [60]. Though some contaminates can, at low levels, actually increase phenoloxidase activity, higher levels reduce activity [60,61,62,63]. The complicated interaction between phenoloxidase activity, temperature, nutrition, and other factors, may explain why we did not detect any effects of urban location measured at a landscape scale.

Honey bee eusociality has resulted in colony-level pathogen defenses as well as individual defenses. Though the worker bees provision, defend, and care for the colony, their reproductive sterility means that there is less pressure on individual survival than colony survival. Honey bees have fewer genes devoted to innate immune responses, and they use the same phenoloxidase for all melanization events [64,65]. Honey bees therefore employ antiseptic (e.g., propolis, GOX activity) and hygienic behaviors (e.g., removal of infected brood) to maintain a sterile colony environment and reduce pathogen pressure and resource costs on all individuals [7]. Though we have shown that the individual immune system shows only minimal effects of urbanization, responses of “social immunity” to urbanization remain unknown. It would also be of great interest to explore the relative effects of these same phenomena across the social gradient of bees, since most species of bees are solitary and therefore are not buffered by mechanisms of social immunity.

Urbanization is a major driver in global change, and urban areas are increasingly considered as experimental systems for studying global warming [66]. Honey bees are a model insect pollinator that can provide insights into how changes in climate and habitat will affect insects and pollination services. Although other research suggests that urbanization and management can affect honey bee pathogen load and diversity [67], our results do not indicate that urbanization negatively affects the immunocompetence of honey bees as measured by these standard bioassays. Importantly, we also found no evidence that there are differences in immunocompetence between managed and feral bees. Thus we demonstrate that honey bee colonies have more phenotypic variation within colonies for important mechanisms of individual immunity than there is among them, and as such external factors of their natural history (urbanization and management) have less of an effect on the physiological expression of disease resistance.
